# A Translational Platform for Polyimide Neural Interfaces: Polyimide Synthesis and in Vivo Evaluation in Epileptic Mice

**DOI:** 10.1109/ACCESS.2026.3674701

**Published:** 2026-03-17

**Authors:** Kshitij Kumar, Kaustubh Deshpande, Naveen Kalur, Garima Chauhan, Deepti Chugh, Subramaniam Ganesh, Arjun Ramakrishnan

**Affiliations:** Department of Biological Sciences and BioengineeringIndian Institute of Technology Kanpur 30077 Kanpur Uttar Pradesh 208016 India; Mehta Family Center for Engineering in MedicineIndian Institute of Technology Kanpur 30077 Kanpur Uttar Pradesh 208016 India; Indian Institute of Technology Bombay Mumbai Maharashtra 400076 India; Eywa Neuro Pvt. Ltd. Mumbai Maharashtra 400086 India

**Keywords:** Neural interfaces, polyimide thin films, microelectrode arrays, MEMS fabrication, biocompatibility, epilepsy monitoring

## Abstract

Thin-film polyimide neural probes have shown great promise in neuroscience but remain difficult to clinically translate due to the unavailability and lack of customizability of commercially available medical-grade polyamic acids. We present an open-source, end-to-end platform for synthesizing BPDA–pPDA-based polyimide from a custom polyamic acid and translating it into microfabricated thin-film neural interfaces. The approach combines accessible polymer chemistry with a streamlined MEMS-compatible fabrication process to produce flexible, biocompatible depth and surface electrode arrays with high thermal stability, chemical inertness, and low moisture uptake. Devices were validated through benchtop characterization, ISO 10993-11 systemic toxicity testing, and in vivo electrophysiology, both acute and semi-chronic, in wild-type and laforin knockout epileptic mice. The arrays reliably captured high-quality multi- and single-unit activity, as well as spontaneous epileptiform discharges, over implantation periods of up to 12 days. By demonstrating a customizable, end-to-end platform for synthesizing and fabricating thin-film polyimide neural electrodes, and by mimicking human neurosurgical workflows through depth, surface, and semi-chronic studies in mice, this work underscores the translational potential of polyimide-based neural microelectrodes and provides a practical pathway to accelerate clinical adoption.

## I. Introduction

The global incidence of neurological disorders and mental health concerns have been rising rapidly, with conditions like epilepsy, Parkinson’s disease, and depression affecting millions worldwide. The World Health Organization (WHO) reports that neurological disorders account for nearly 6.3% of the global disease burden, with epilepsy alone affecting around 50 million people globally. Similarly, mental health disorders, including depression, have become leading causes of disability, significantly contributing to the global health burden (WHO, 2021). While pharmacological treatments remain the primary therapy, a substantial percentage of patients—up to 30% in the case of epilepsy—are resistant to drug treatments [1]. In these drug-resistant cases, implantable neural devices, such as deep brain stimulation systems, vagus nerve stimulators (VNS), and cortical implants, have demonstrated clinical success in providing therapeutic relief.

These devices have been particularly effective in managing conditions such as epilepsy, depression, and Parkinson’s disease, especially when traditional pharmacological therapies fail. For instance, DBS has been widely adopted for treating Parkinson’s disease, while responsive neurostimulation (RNS) has proven to be an effective solution for epilepsy patients with drug-resistant seizures [2]. However, the cost of these procedures and devices places them beyond the reach of many, particularly in low- and middle-income countries (LMIC) where healthcare resources are limited. This underlines the urgent need for affordable, scalable, and reliable neural implants that can broaden access to these life-saving technologies.

MEMS-based thin film technology offers promising solutions to some of these challenges. It has driven advancements in neural interfaces by enabling device miniaturization and the development of high-channel-count arrays that are less invasive to brain tissue. Traditional materials like silicon and metal microwires, although providing excellent signal-to-noise ratios, have drawbacks such as stiffness, brittleness, and biocompatibility concerns, limiting their broad clinical use [3], [4], [5]. In contrast, flexible substrates like polyimide, parylene, and liquid crystal polymer align more closely with the mechanical properties of brain tissue, reducing foreign body reactions and improving long-term stability [6], [7]. While comparisons to silicon and metal microwires provide historical context, more relevant benchmarks for flexible neural interfaces are other polymeric substrates such as parylene C [8], [9], [10], [11] and SU-8 [12], [13]. Parylene C has been widely used due to its conformal coating capability, chemical inertness, and established biocompatibility; however, it has a limited thermal budget which limits its versatility in microfabrication. SU-8, an epoxy-based negative photoresist, offers high resolution and mechanical stability but is inherently brittle [14], and has limited flexibility [15]. SU-8 is not recommended for in vivo applications, and, it has not been incorporated into any FDA-approved implantable medical device. Polyimide is especially notable for its dielectric properties, flexibility, and thin-film fabrication potential, making it a popular choice for neural interfaces [16], [17]. However, the limited availability of medical-grade polyimide precursors continues to hinder the widespread clinical adoption of MEMS-based thin film neural interfaces for acute neurosurgical applications. Several commercial and noncommercial polyimide (PI) formulations have been demonstrated to be non-toxic through a range of in vitro cytotoxicity assays, including both direct and indirect evaluations. These studies have examined key cellular responses such as viability, proliferation, degeneration, and lysis across multiple commonly used cell types [18], [19], [20], [21], [22]

Polyimide thin films are synthesized from polyamic acid, which undergoes spin coating and high-temperature curing to convert into polyimide through the imidization process [23], [24]. Commercially available polyamic acids, such as Pyralin 2610/2611 by HD Microsystems and U-Varnish by UBE Industries, are commonly used in electronic applications but are generally not approved for human medical use [25]. The specific properties of polyimide, determined by the monomers used in its synthesis, particularly biphenyl dianhydride (BPDA) and p-phenylenediamine (p-PDA), are crucial for its biocompatibility and stability [23]. Despite its potential, the scarcity of medical-grade polyimide limits its use in human clinical settings.

One of the critical applications of neural electrodes is in epilepsy monitoring, where stereotactic electroencephalography (sEEG) and depth electrodes are used to detect and monitor seizures in patients with drug-resistant epilepsy. Accurate neural recordings from depth electrodes are essential for identifying epileptic foci and guiding surgical interventions. Flexible and biocompatible electrodes, such as those made from polyimide, offer significant advantages, as they reduce tissue damage and foreign body responses during long-term implantations. Extended monitoring in animal models, like the laforin knockout mice, provides valuable insights into disease progression and potential treatments [26], [27].

Commercial polyamic acids and polyimide formulations used for microfabrication, such as PI-2611 (HD Microsystems) and U-Varnish-S (UBE Industries), are proprietary materials with undisclosed chemistries. As a result, researchers and early-stage companies have limited ability to tune material parameters such as viscosity, molecular weight, or solids content, and independent certification of chemical safety for implantable use is challenging. The goal of this work is therefore not to outperform these materials, but to provide an open and reproducible synthesis route for BPDA–pPDA polyamic acid that yields polyimide films with properties desirable for neural microelectrodes, while enabling customization and local manufacturing.

In this study, we address critical limitations in translating MEMS-based microelectrode technologies to acute neurosurgical interventions like microelectrode recordings (MER) and sEEG. By developing a custom polyamic acid chemistry, we demonstrate its application in the microfabrication of neural interfaces, enabling MEMS technology’s full potential, such as channel scaling and flexibility for human neurosurgical applications, particularly in epilepsy monitoring. The following objectives were achieved: 1. Synthesis of BPDA-pPDA polyimide. 2. Characterization of surface roughness, thermal stability, chemical inertness, and insulation properties of the polyimide film. 3. Microfabrication of 4 to 32-channel, $10~\mu $m thick, depth and surface neural microelectrode arrays. 4. Benchtop electrochemical characterization of the electrode-electrolyte interface. 5. Passing ISO 10993-11 acute systemic toxicity tests for synthesized polyimide thin films. 6. Surface and intracortical implantation of the electrode arrays. 7. Demonstration of high-quality neural activity during acute surgeries, including isolated single units and quantified signal-to-noise ratios in a healthy control as well as a rodent model of epilepsy (laforin knockout). 8. Semi-chronic implantation of tetrodes (up to 12 days) in the laforin knockout mice to register epileptic seizures.

## II. Materials and Methods

### A. Polyamic Acid Synthesis and Characterization

Polyamic acid (PAA) was synthesized via equimolar polycondensation of BPDA (Merck, India) and p-PDA (Merck, India) in dimethylacetamide (DMAc, Leo Chemo Plast Pvt. Ltd, India). The reaction was carried out in a nitrogen-purged, four-necked flask equipped with a condenser, agitator, and thermocouple. First, 250 g of DMAc was heated to 30 °C, followed by dissolution of 0.078 moles of p-PDA at 90 °C for 2 h. After cooling to 50 °C, an equimolar amount of BPDA was added and stirred for 3 h, then cooled below 30 °C and stirred for an additional 7–8 h to form a viscous yellow solution. The product was vacuum-filtered (Whatman Grade 41) and degassed under vacuum for 24–48 h.

Thermal and curing behavior were assessed via thermogravimetric analysis (TGA) and differential scanning calorimetry (DSC) (N = 3). Fourier-transform infrared spectroscopy (FTIR) (N = 5) was used to confirm imidization, and to show disappearance of amide peaks and emergence of characteristic imide bands. Chemical inertness was evaluated by encapsulating metal traces in polyimide and exposing them to cleanroom chemicals; electrochemical impedance spectroscopy (EIS) in PBS verified insulation integrity. Surface quality was measured by atomic force microscopy (AFM) over 2 mm $\times 2$ mm areas, yielding sub-nanometer roughness. Moisture uptake was determined by weighing cured films before and after 24 h water immersion, followed by drying at 100 °C for 10 min in the TGA microbalance.

### B. Microfabrication

**FIGURE 1.**
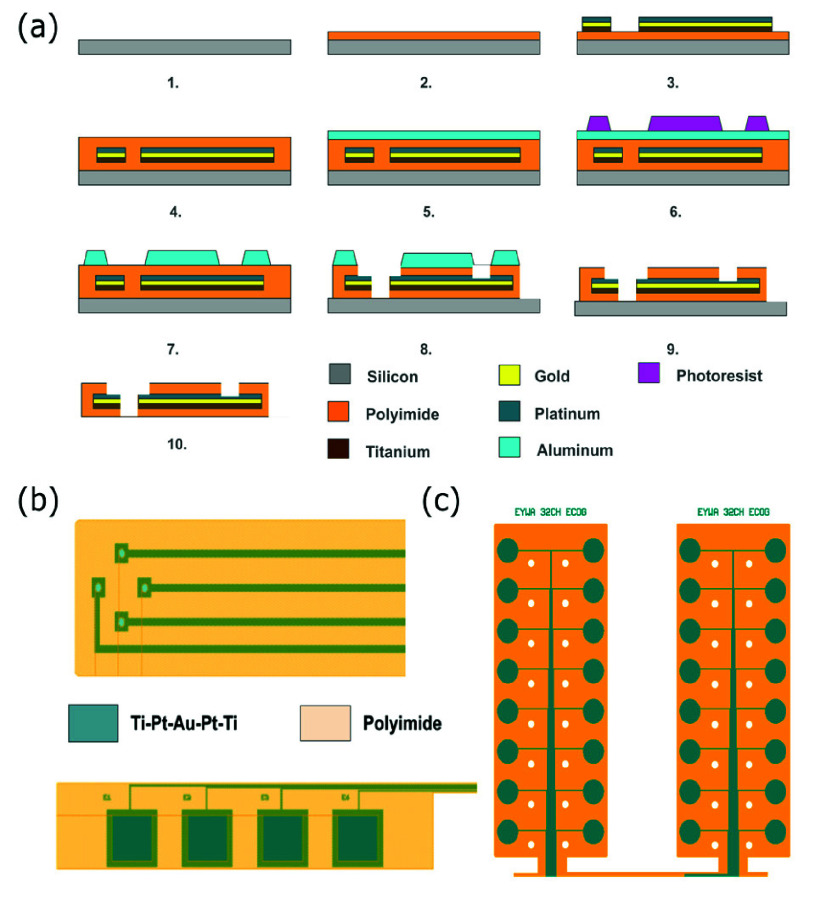
(a) Microfabrication process flow for polyimide-based neural recording interfaces [Steps (1-2) Polyimide spin coating and roughening, Steps (3-4) Metal deposition and liftoff, Steps(5-7) Aluminium hard mask deposition and patterning, Steps(8-9) O_2_ plasma etching and Al hard mask removal Step(10). Device release] (b) Electrode designs for tetrode depth and surface arrays, along with 32 channel surface probes.

Neural probes were fabricated using a two-mask process on RCA-cleaned silicon wafers as shown in Fig 1a. A base polyimide layer was formed by spin-coating synthesized polyamic acid at 5000 rpm, soft-baking at 100 °C for 5 min and 120 °C for 5 min, followed by curing in nitrogen at 350 °C, with 30 min isothermal intervals at 150°C and 250°C and a ramp rate of 4°C/min. The cured surface was roughened with O_2_ plasma (PDC-32G-2, Harrick Plasma Systems) to enhance adhesion.

Metal traces were patterned using a bilayer lift-off process with S1813 photoresist over LOR3B. A Ti (20 nm)/ Pt(50nm)/ Au (200 nm) / Pt (50 nm)/ Ti(20nm) stack was deposited by sputtering (AJA Systems) and lifted off in Remover PG, yielding $7~\mu $m-wide features. Residual resist was removed via O_2_ plasma descum.

A second polyimide layer was spin-coated and cured to achieve a total thickness of $10~\mu $m. Electrode and bondpad openings were defined using a 100 nm sputtered Al hard mask, patterned in S1813 and wet-etched (Type A aluminum etchant, Transene). The polyimide was etched with O_2_ plasma (PDC-32G-2, Harrick Plasma Systems) to expose electrode sites and bondpads, after which the Al mask was stripped. Devices were released from wafers by soaking in isopropanol and manually peeling with fine tweezers. The neural interfaces used through hole pads with exposed metal rims that were bonded to a custom adaptor made to mate directly with an Intan RHS head stage.

### C. Electrode Design and Impedance Characterization

Electrode geometries (Fig 1b.) were tailored for murine brain anatomy and surgical handling. Depth probes consisted of 5 mm-long, $80~\mu $m-wide shanks carrying four $20\times 20~\mu $m platinum recording sites spaced $150~\mu $m center-to-center. Four-channel Surface ECoG arrays featured $800\times 800~\mu $m sites with 1.5 mm inter site spacing on a 7.5 mm $\times 1$ mm polyimide substrate. Custom 32-channel Surface ECoG arrays incorporated $500~\mu $m-diameter sites on two 8.5 mm $\times 3.3$ mm flaps for bilateral cortical coverage.

Impedance characterization was performed using a Metrohm STAT-I-400S potentiostat in $1\times $ phosphate-buffered saline (PBS) with a three-electrode configuration: Ag/AgCl reference electrode (BASi MF-2052), 0.5 mm-diameter platinum wire counter electrode, and the fabricated device as the working electrode. Measurements were acquired from 1 Hz to 10 kHz using a 50 mV sinusoidal excitation, with three points per frequency decade.

### D. Animal Models and Surgical Protocol

Surgical procedures were approved by the Institutional Animal Ethics Committee (IAEC Project No. IITK/IAEC/ 2021/1137). C57BL/6 mice and transgenic C57BL/6 laforin knockout mice were used across multiple recording sessions. The wild-type mice served as controls, while the laforin knockout mice—a validated model of drug-resistant epilepsy—enabled evaluation of probe performance in detecting spontaneous epileptiform activity [26].

To stiffen the flexible electrode tip before implantation, tetrode depth probes were dip-coated with polyethylene glycol (PEG, P2906, Sigma-Aldrich, USA; melting point 50–60 °C). Mice were anesthetized with isoflurane (5% induction, 1–1.5% maintenance) in 100% oxygen. Anesthesia depth was monitored using the toe-pinch withdrawal reflex and re-evaluated every 10–30 min. A heating pad was used for thermoregulation. Lidocaine (0.1 mg/kg) was administered subcutaneously for local anesthesia.

A primary incision was made along the midline, and bregma was located using a surgical microscope. A $1\times 1$ mm craniotomy was performed with a micro-drill, taking care not to damage the dura or blood vessels. A 5 mm-long, 4-channel polyimide depth probe ( $20\times 20~\mu $m Pt sites) was implanted into the somatosensory cortex (AP: −0.9 mm, ML: + 3 mm, DV: −1.5 mm) [28]. A 4-channel Surface ECoG array (7.5 mm length, $800\times 800~\mu $m electrode area) was placed over the left cortex using a robotic stereotaxic arm (Neurostar, GMBH, Germany). For whole-brain recordings, a 32-channel custom Surface ECoG array (8.5 mm length, 3.3 mm width per flap, $500~\mu $m site diameter) was positioned to cover both hemispheres.

For semi-chronic recordings, probes were implanted for up to 12 days, with recordings carried out for up to 10 days post-implantation following a 3-day recovery period. This duration aligns with typical clinical stereo-EEG (sEEG) monitoring timelines in drug-resistant epilepsy, where electrode implantation commonly spans 7–11 days [29], [30], [31], [32], providing a clinically relevant time frame for assessing electrode stability and recording quality, although longer implant durations are warranted sometimes.

### E. In-Vivo Recordings and Neural Data Analysis

Electrophysiological data were acquired using the Intan RHS stim/recording system (Intan Technologies) inside a custom Faraday cage. The RHS32 low-noise amplifier headstage digitized signals at 30 kHz. A 50 Hz notch filter was applied in real time to remove electrical noise, and a 300–6000 Hz bandpass filter was used for neuronal activity monitoring.

Data were analyzed in Python (v3.10) using the SpikeInterface library (v0.100). Wideband neuronal signals were re-referenced using central median referencing and bandpass-filtered between 300–6000 Hz. Signals were Z-score normalized and whitened before spike sorting with the Mountainsort5 (MS5) algorithm [33].

For single-unit activity (SUA), clusters with an interspike interval (ISI) ratio <0.5 were classified as SUA; all clusters were included for multi-unit activity (MUA). For Surface ECoG recordings, only MUA was analyzed. For each recording day, the maximum number of neuronal clusters detected in any session was reported. Signal-to-noise ratio (SNR) was averaged across sessions for the day.

### F. Biocompatibility Testing

Given the wide diversity of polyimide formulations, each variant requires independent evaluation to determine its biological safety. Even small variations in monomer composition, molecular weight, or curing chemistry can lead to significant differences in cytotoxicity, thrombogenicity, tissue interaction, and long-term biostability [34], [35].

To evaluate the systemic safety of the synthesized polyamic acid and its corresponding polyimide films, acute systemic toxicity testing (ISO 10993-11:2017) was performed. This assessment was chosen as the most appropriate materials-level evaluation [36], as it determines whether the synthesis and curing processes produce any toxic or leachable compounds that could cause systemic adverse effects. The test directly validates the chemical safety of the proposed synthesis route, ensuring that the polymer and its processing steps do not introduce harmful byproducts. According to ISO 10993-1:2018 and the U.S. FDA’s guidance [37] on the use of ISO 10993-1 for biological evaluation of medical devices (FDA, 2023), other biological evaluations, such as cytotoxicity, irritation, sensitization, implantation, and local tissue response, are intended for finished medical devices produced under GMP or cleanroom manufacturing conditions, where geometry, surface finish, sterilization, and intended use influence biological outcomes. These device-level assessments were therefore beyond the scope of this materials-focused study, which aimed to establish a reproducible and biocompatible framework for polyimide film synthesis suitable for future device development.

Biocompatibility was evaluated according to ISO 10993-11 guidelines for acute systemic toxicity. Testing was performed at RCC Laboratories, Hyderabad, an accredited facility for preclinical safety assessments, under OECD GLP compliance and NGCMA certification.

Twenty male Swiss Albino mice (Mus musculus; 6–7 weeks; 18.91–20.69 g) were randomized into four groups (N = 5/group): Group 1 (polar blank, intravenous), Group 2 (polar test extract, intravenous), Group 3 (non-polar blank, intraperitoneal), and Group 4 (non-polar test extract, intraperitoneal). Extracts were prepared from the fabricated polyimide films, confirmed free of particulates post-autoclaving, and dosed at 50 mL/kg via the USP-recommended routes.

Clinical observations including mortality, viability, and clinical signs, were conducted immediately post-dosing and at 4, 24, 48, and 72 h. Body weights were recorded during acclimatization, pre-treatment, and pre-necropsy. Necropsy involved macroscopic examination of injection sites and major organs.

## III. Results and Discussion

### A. Polyimide Synthesis, Characterization, and Microfabrication

The synthesized BPDA–pPDA polyamic acid exhibited an inherent viscosity of 2.0 dL/g (Ubbelohde viscometer), indicating a polymer chain length suitable for stable thin-film formation. Spin-coated films on 50 mm silicon wafers were soft-baked at 100 °C for 5 min and 120 °C for 5 min, producing uniform coatings with no pinholes or contamination under optical microscopy, confirming formulation stability during spin coating and baking.

The proposed synthesis route is cost-efficient and inherently scalable, as microfabrication requires only gram-scale quantities of polymer. Using readily available precursors and standard laboratory equipment, the process can be economically implemented in academic settings and proportionally scaled for larger production. This framework therefore reduces reliance on commercial suppliers and enhances the availability and customizability of polyimide, enabling researchers to tailor material properties to specific neural interface designs while supporting broader adoption and innovation in the field.

Using a two-mask MEMS process, $10~\mu $m-thick depth and Surface ECoG arrays were fabricated. The process involved sequential polyimide coating and curing, Ti/Pt/Au/Pt/Ti metal stack deposition (AJA sputter system), O_2_ plasma roughening, Al hard mask patterning, and O_2_ plasma etching to define electrodes and bondpads. Released devices were flat and free of distortions, demonstrating mechanical stability.

**FIGURE 2.**
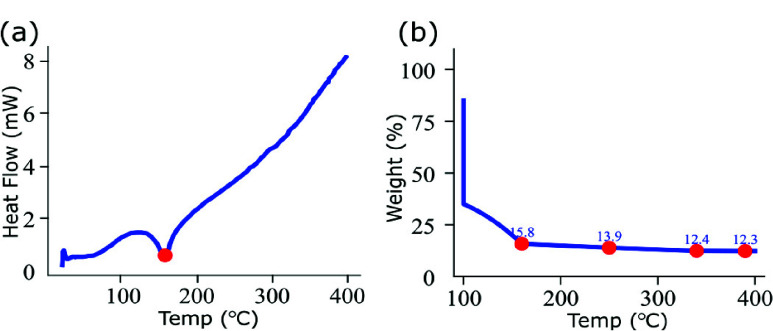
Thermal analysis of the synthesized BPDA–pPDA polyamic acid. (a) Differential scanning calorimetry (DSC) curve showing a single endothermic peak near 160 °C corresponding to solvent evaporation and stable heat flow up to 400 °C. (b) Thermogravimetric analysis (TGA) curve indicating weight loss between 160–350 °C due to imidization, with minimal change beyond 350 °C.

DSC thermograms (Fig. 2a), recorded during heating to 400 °C at 10 °C/min, showed a single event at ~160 °C, attributed to DMAc solvent evaporation, with steady non-reversing heat flow up to 400 °C. TGA (NEXTA STA300, Hitachi) of the polyamic acid with isothermal holds at 150 °C, 250 °C, and 350 °C revealed continuous weight loss from 160 °C to 350 °C due to cyclization and imidization, with residual mass changes of 13.9% (250 °C), 12.4% (350 °C), and 12.3% (400 °C), indicating near-complete imidization above 350 °C (Fig. 2b).

**FIGURE 3.**
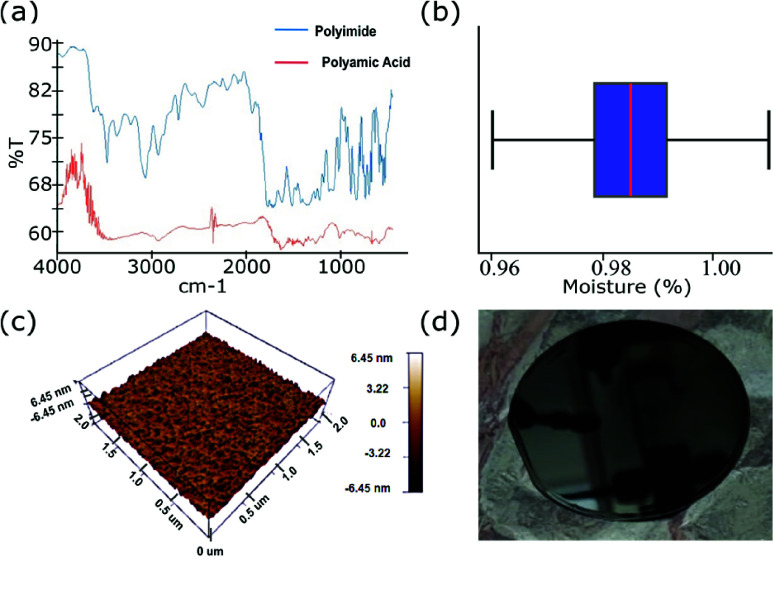
Bulk and surface properties of spun BPDA–pPDA polyimide films. (a) FTIR spectra showing disappearance of amide peaks and appearance of imide peaks, confirming complete imidization. (b) Moisture uptake measured using a TGA microbalance ( $N =6$). (c) AFM topography (2 mm $\times 2$ mm scan) of a $5~\mu $m film showing a uniform surface with average roughness of ~0.56 nm. (d) Polyamic acid was spin-coated on 50 mm silicon wafers and soft-baked on a hotplate at 100 °C for 5 minutes followed by 120 °C for 5 minutes. The spun wafers were then inspected under high magnification on an optical microscope for the presence of any pinholes or particles.

FTIR (Vertex 80, Bruker) confirmed imidization by the disappearance of the N–H stretch and amide I peaks (1653 cm^−1^) and the emergence of imide peaks: C = O stretch at 1768 cm^−1^, C–N stretch at 1378 cm^−1^, C–H bend at 1123 cm^−1^, and C = O bend at 736 cm^−1^, consistent with prior polyimide studies [38] (Fig. 3a).Moisture uptake tests on six cured films, after 24 h immersion in deionized water and drying at 100 °C, showed a mean absorption of ~0.98%, indicating minimal hygroscopic degradation (Fig. 3b). AFM (MFP3D Origin, Oxford Instruments) scans over 2 mm $\times 2$ mm areas of $5~\mu $m films yielded an average surface roughness of 561.43 pm (min: −1.31 nm, max: 1.6 nm), meeting sub-nanometer requirements for high-resolution lithography (Fig. 3c).

Chemical resistance testing on polyimide-encapsulated metal lines immersed for 48 h in PRS 2000, Type A aluminium etchant, $1\times $ PBS, HCl, and IPA showed no impedance change (EIS, 100 Hz–100 kHz) or visual damage, confirming suitability for cleanroom processing.

**FIGURE 4.**
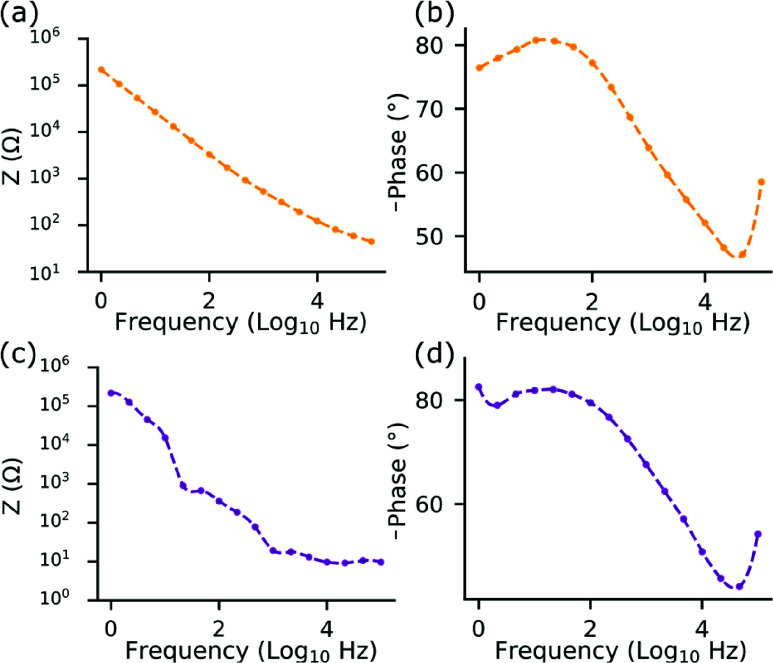
Electrochemical impedance spectroscopy of fabricated neural arrays. (a, b) Impedance magnitude and phase for depth electrodes. (c, d) Impedance magnitude and phase for 4-channel Surface ECoG arrays measured in 1x PBS using a three-electrode setup with a 50 mV excitation.

Electrochemical performance was characterized by EIS using a Metrohm STAT-I-400S potentiostat in $1\times $ PBS with a three-electrode configuration (Ag/AgCl reference, 0.5 mm Pt counter, device as working electrode). At 1 kHz, depth electrodes ( $400~\mu $m^2^ sites) exhibited an average impedance of 586.1 ± 15.2 k $\Omega $ (Fig. 4a–b) for N = 5 arrays, while a 4-channel Surface ECoG array (0.64 mm^2^ sites) exhibited an average impedance of 18.21 ± 0.7 k $\Omega $ for N = 5 arrays (Fig. 4c-d). Impedance spectra showed capacitive behavior at low frequencies, transitioning to resistive behavior at higher frequencies, consistent with electrochemical characteristics required for multi-unit and single-unit recordings.

The synthesized BPDA–pPDA polyamic acid satisfied the key requirements for producing thin films suitable for high-performance neural interfaces. Thermal, surface, and chemical characterization confirmed compatibility with high-temperature MEMS processing, environmental stability, and robustness against standard cleanroom chemicals. While this formulation can be customized by varying inherent viscosity to yield thinner or thicker films as needed and can be further modified to incorporate additional functionalities, such as photopatternable compounds, to expand fabrication versatility. Rather than aiming to outperform proprietary commercial polyimides, the goal of this work is to provide an open and reproducible synthesis route that enables such tunability, which is not possible with undisclosed commercial formulations. Direct numerical cost comparison with commercial polyamic acids is not feasible due to proprietary pricing and regional variability; however, the use of commodity monomers and standard laboratory equipment enables low-volume in-house synthesis and scalable local production. The resulting depth and Surface ECoG arrays demonstrated impedance characteristics and frequency-dependent behavior consistent with high-quality neural recording electrodes, matching reported benchmarks in the literature [5], [39]. With a focus on translational application and open-source accessibility for neural engineers, this platform can serve as a fully customizable pathway for translating thin-film neural devices from the research bench to human clinical use.

### B. Acute Neural Recordings Using Polyimide Probes

**FIGURE 5.**
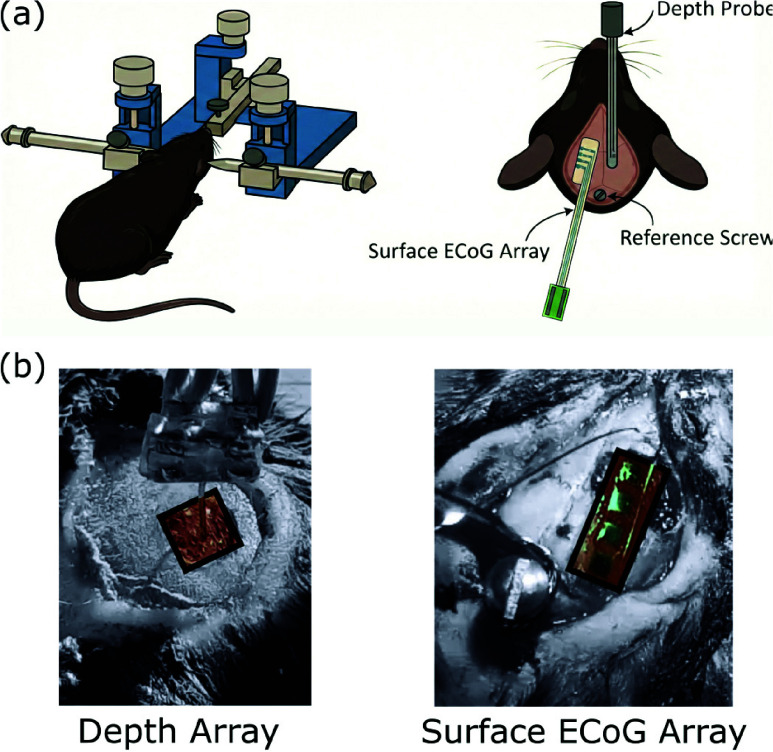
Surgical implantation and positioning of neural recording arrays in an mouse model. (a) Illustrated image of an anesthetized mouse mounted on a stereotactic frame showing placement of the depth tetrode and Surface ECoG arrays. (b) Intraoperative images showing electrode placement on the cortical surface for neural recordings.

Probe geometries and animal models were selected to assess performance under healthy and epileptic conditions. The 4-channel depth tetrodes and Surface ECoG arrays were dimensioned for murine brain anatomy to target the somatosensory cortex with minimal trauma, while wild-type (WT) mice served as controls and laforin knockout (LKO) mice provided a validated model of spontaneous seizures [26]. Acute recordings were performed using 4-channel depth tetrodes, 4-channel Surface ECoG arrays, and 32-channel Surface ECoG arrays. Figure 5 shows the surgical setup and probe configurations, including the stereotaxic placement of the depth and surface arrays, and the assembled 32-channel Surface ECoG array connected to the recording headstage.

**FIGURE 6.**
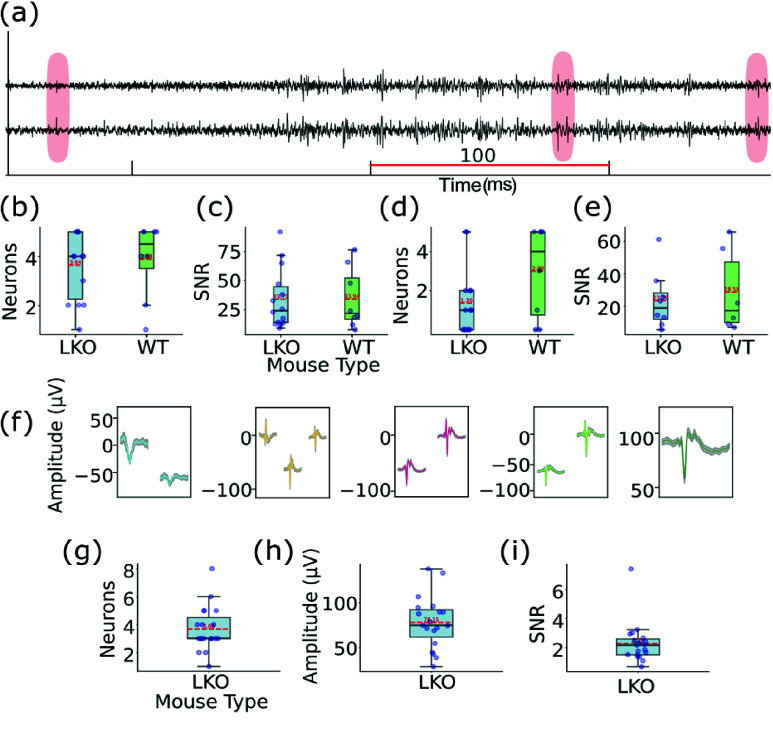
Quality of acute neural recordings from 4-channel depth and surface arrays in wild-type (WT) and laforin knockout (LKO)mice. (a–f) Depth tetrode recordings showing representative raw traces from two channels of an implanted probe in an LKO mouse, with neuronal spikes highlighted; average multi-unit activity (MUA) across sessions in WT (green, N = 8) and LKO (blue, N = 14) mice;signal-to-noise ratio (SNR) comparison for MUA and single-unit activity (SUA); representative sorted SUA waveforms demonstrating single-unit isolation. (g–i) Surface ECoG recordings from LKO mice (N = 2) showing average MUA, amplitude across sessions, and corresponding SNR, confirming stable population-level activity.

Depth probes in LKO mice captured high-frequency spiking activity (Fig. 6a) and well-isolated single-unit clusters (Fig. 6f). Multi-unit activity (MUA) in LKO mice yielded 3.64 ± 0.37 neurons per recording (SNR: 33.75 ± 6.95; presence ratio: 1.00 ± 0.00), comparable to WT mice (3.88 ± 0.55 neurons; SNR: 33.94 ± 9.20; presence ratio: 0.96 ± 0.04). Single-unit activity (SUA) analysis showed 1.36 ± 0.46 neurons in LKO (SNR: 23.29 ± 6.45) and 3.00 ± 0.82 neurons in WT (SNR: 28.58 ± 10.49), with presence ratios exceeding 0.83.

For surface recordings, 4-channel surface ECoG arrays in LKO mice detected 4.50 ± 0.50 neurons (MUA) with an SNR of 4.81 ± 2.61, a presence ratio of 1.00 ± 0.00, and a firing rate of 18.31 ± 4.08 Hz (Fig. 6g–i). As expected, SNR was lower than for depth recordings, but the arrays provided broader cortical coverage with minimal tissue disruption. Together, these results confirm that the fabricated probes enable high-fidelity neuronal recording and single-unit isolation in both healthy and epileptic models.

The acute recordings demonstrate that the fabricated BPDA–pPDA polyimide probes achieve electrical performance and neural signal quality comparable to established flexible electrode platforms. The high SNR and consistent presence ratios observed in both WT and LKO mice confirm stable electrode–tissue interfaces during short-term implantation. The ability to isolate SUA in both models, including under epileptiform activity in LKO mice, highlights the probes’ suitability for applications requiring precise single-unit resolution. The lower SNR observed in Surface ECoG arrays compared to depth probes is consistent with the expected attenuation from non-penetrating electrodes but is offset by their larger cortical coverage and reduced invasiveness. These findings validate the probe designs for both acute functional mapping and seizure detection studies, supporting their use as a translational tool for preclinical testing of flexible thin-film neural interfaces.

### C. Semi-Chronic Neural Recording and Epilepsy Monitoring Using Polyimide Probes

**FIGURE 7.**
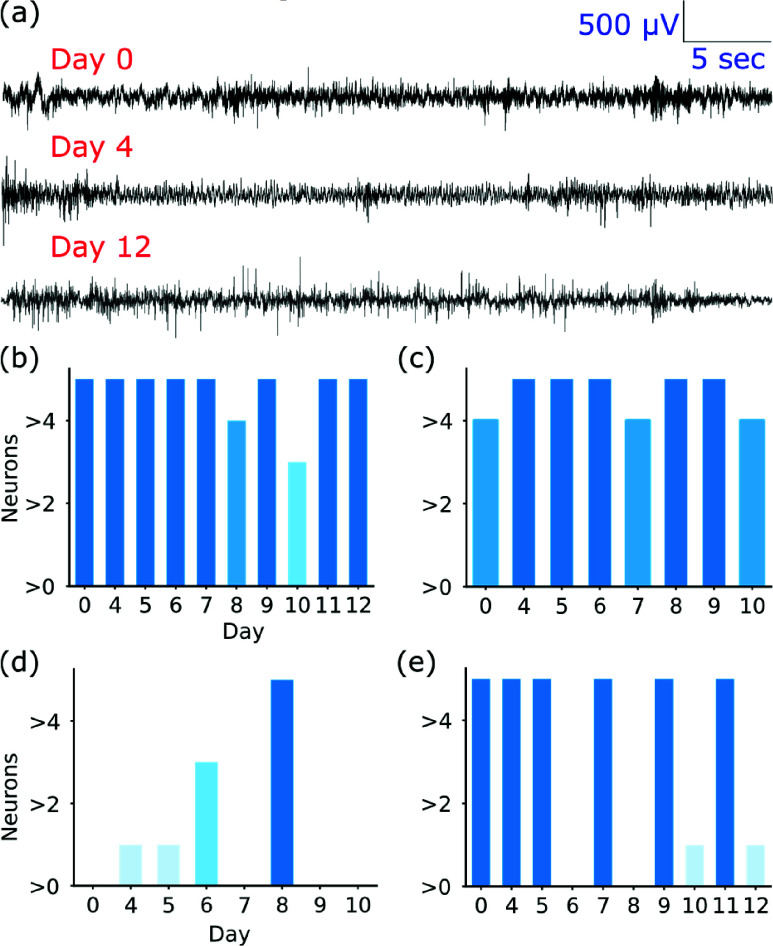
Longitudinal neural recordings up to 12 days in laforin knockout(LKO) C57BL/6 mice. (a) Representative neural traces from semi-chronically implanted depth probes at days 0, 4, and 12, showing changes in signal characteristics over time.(b–e) Quantitative analysis of multi-unit (MUA) and single-unit(SUA) activity for two animals (N = 2) across the 12-day period. The y-axis indicates neuron count, the x-axis represents recording days, color intensity reflects neuronal density, and error bars denote the standard error of the mean from multiple ~1-hour sessions per day.

Semi-chronic recordings (Fig. 7a) were conducted to assess the short-term stability of the fabricated probes in an epileptic model. Laforin knockout (LKO) mice, which exhibit spontaneous recurrent seizures and serve as a well-established model of drug-resistant epilepsy [26], were used to evaluate the probes’ ability to capture epileptiform activity over extended periods, simulating the continuous monitoring required in stereo electroencephalography (sEEG).

Two LKO mice were implanted with 4-channel depth tetrodes for up to 12 days, and recordings were obtained for 10 days following a 3-day recovery period. In Mouse 1, MUA SNR remained stable across sessions (Day 0: 7.89 ± 0.48; Day 4: 8.70 ± 1.49; Day 10: 8.14 ± 0.88) with presence ratios above 0.96. Mouse 2 exhibited SNR values of 11.00 ± 1.10 (Day 0), 17.19 ± 2.87 (Day 4), and 7.19 ± 0.05 (Day 12), with presence ratios improving from 0.60 ± 0.08 to 0.91 ± 0.04 (Fig. 7b–c). SUA analysis reveals strong early post-surgical signals (Mouse 1, Day 4: SNR 55.00; presence ratio 1.00 ± 0.01), followed by a gradual decline from Day 8–12, consistent with expected tissue response and electrode drift (Fig. 7d–e).

**FIGURE 8.**
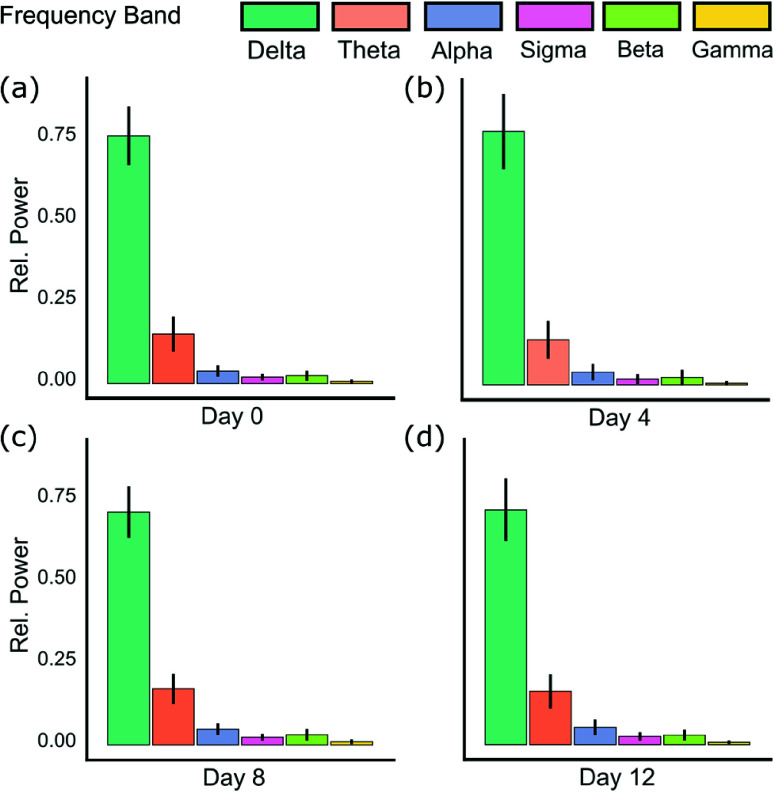
Relative spectral power across Days 0, 4, 8, and 12 remained stable. Variability was low across delta–low gamma bands (0.5–80 Hz), with coefficients of variation below 17% and particularly strong consistency in delta and theta bands(<6%).

Signal degradation is a universal challenge in chronically implanted neural interfaces, arising from glial encapsulation and impedance changes at the electrode–tissue interface [40]. However, low-frequency local field potentials (<200 Hz), critical for seizure monitoring, are typically more resilient to degradation than high-frequency signals. Our spectral analysis across Days 0, 4, 8, and 12 confirmed this stability: delta through low-gamma bands (0.5–80 Hz) showed coefficient of variation <17%, with delta and theta bands (<6%) demonstrating consistency within established EEG test–retest reliability bounds [41], as depicted in Fig. 8.

**FIGURE 9.**
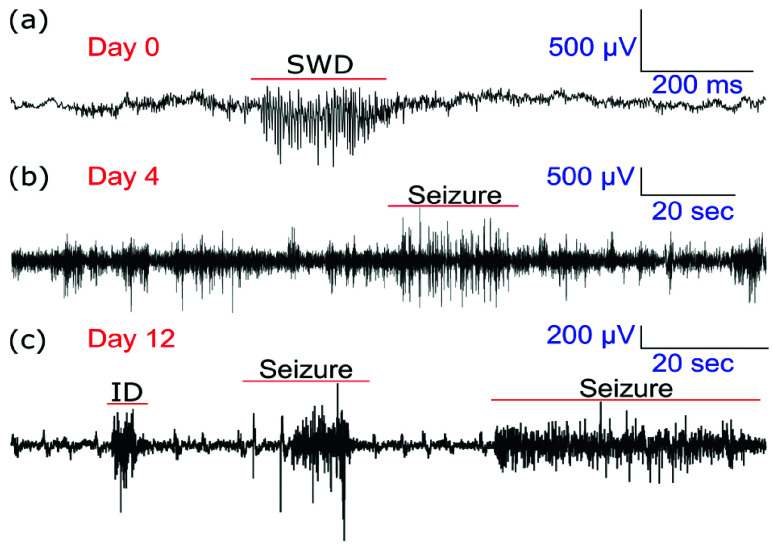
Epileptiform activity patterns recorded from depth probes in laforin knockout (LKO) C57BL/6 mice during semi-chronic implantation. Representative traces from the same animal on (a) day 0, (b) day 4, and (c) day 12 demonstrate stable post-surgical recordings capturing epileptiform discharges.(SWD: spike-wave discharge; ID: interictal discharge.)

As can be seen in Fig. 9, epileptiform discharges, including spike-wave patterns and prolonged ictal events, were detected as early as Day 0 and persisted throughout the implantation period.

These findings together demonstrate that our electrodes maintain clinically relevant recording fidelity throughout typical sEEG implantation periods.

Although this implantation duration does not represent long-term chronic use, it reflects clinically relevant monitoring timelines for sEEG procedures and demonstrates stable neural recording performance over the intended short-term use window.

### D. Bilateral Surface Recording with 32 Channel ECoG

**FIGURE 10.**
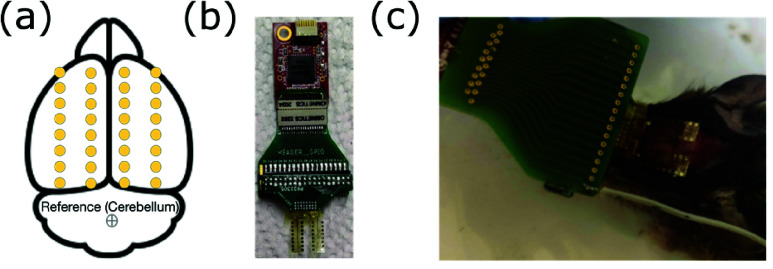
High-density surface mapping of epileptiform activity using a custom 32-channel ECoG array. (a) Array layout showing electrode positions for cortical mapping of epileptiform discharges and seizure foci. (b) Fully assembled 32-channel Surface ECoG array connected to an Intan RHS headstage. (c) Array placement on the mouse brain.

To expand cortical coverage, we developed a custom 32-channel electrocorticography (ECoG) array designed for bilateral surface recordings from the mouse cortex (Fig. 10). To demonstrate the spatial mapping capabilities of the 32-channel array, recordings were obtained from an LKO mouse during spontaneous epileptiform activity. The flexible, polyimide-based array conforms to the cortical surface, providing stable contact as demonstrated in Fig. 10(a,c) and allowing simultaneous detection of epileptiform discharges across multiple cortical regions.

The array’s broad spatial footprint supports bilateral recordings across both hemispheres (Fig. 10a.), enabling us to detect and map transient ictal discharges that were present across both cortical hemispheres (Fig. 10c.). These cross-hemispheric seizure events could provide critical insights into the distributed network mechanisms that underlie generalized seizures and interregional synchrony in epilepsy.

The primary goal of our initial report on the 32-channel ECoG recordings was to demonstrate that our custom-designed array provides broad spatial coverage of the mouse cortical surface, thereby enabling high-resolution spatial localization of cortical activity.

**FIGURE 11.**
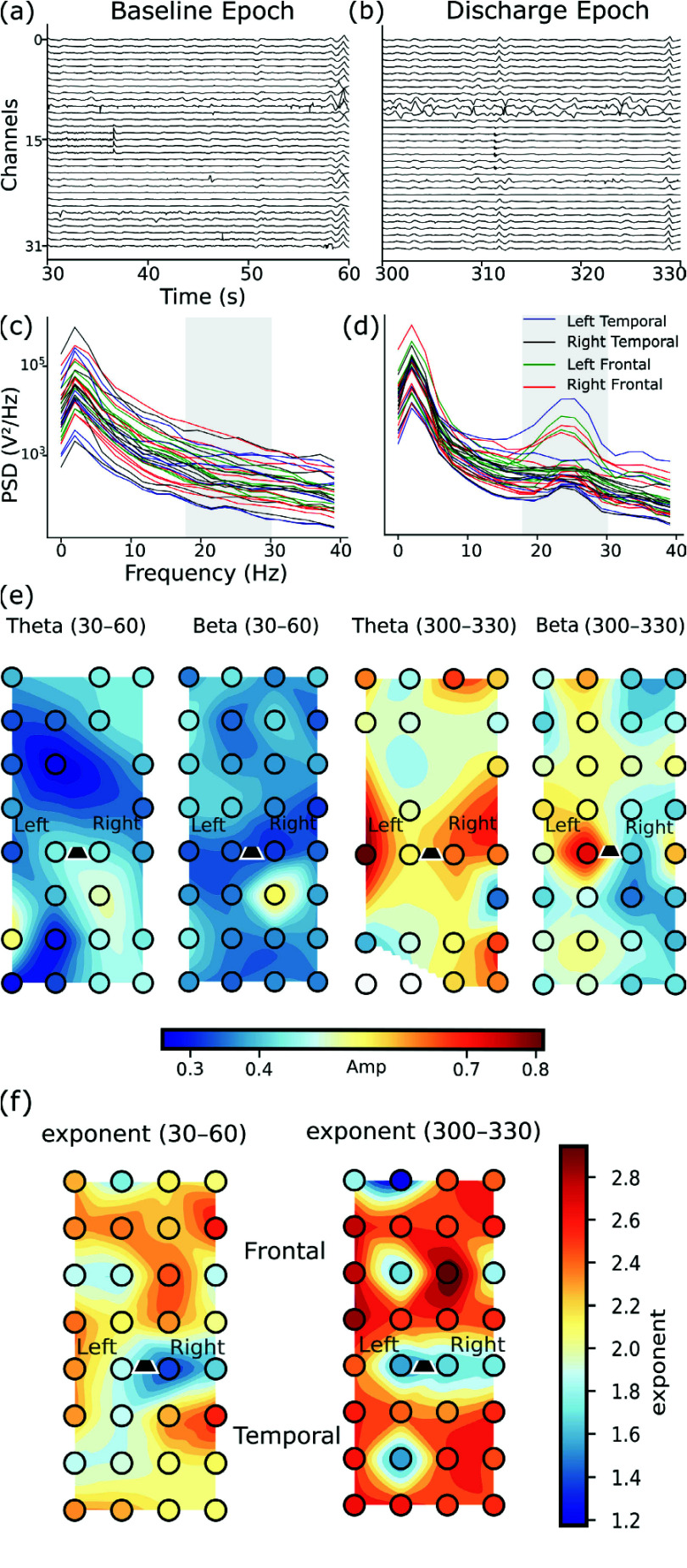
Spatial mapping capabilities of the 32-channel ECoG array demonstrated in one LKO mouse. Representative recording from an LKO mouse demonstrating the array’s capability to resolve spatiotemporal patterns of cortical activity. The raw neural traces for the baseline segment and the discharge segment are shown in (a, b). Power spectral density (PSD) and topographic analysis of neural oscillations: (c) PSD for 30-60s time window showing spectral power across frequencies (0-40 Hz) for different brain regions (left temporal, right temporal, left frontal, right frontal), with highlighted beta band (18-30 Hz). (d) PSD for 300-330s time window displaying increased beta band activity compared to earlier timepoint. (e) Topographic maps of theta(4-8 Hz) and beta (18-30 Hz) band average amplitude across electrode array positions for 30-60s and 300-330s windows, showing frontal and temporal spatial distribution patterns. (f) Topographic maps of power spectral exponent values across electrode positions for 30-60s and 300-330s windows, showing frontal-to-temporal gradient with higher exponents (warmer colors) in frontal regions and lower exponents (cooler colors) in temporal regions.

As proof-of-concept to demonstrate the array’s spatial resolution, we performed preliminary spatial analysis on two representative 30-second epochs from a single LKO mice model: (i) a baseline segment (30-60s) and (ii) a segment exhibiting an abnormal discharge (300-330s) (Fig. 11c, d). The spatial analysis illustrates the array’s capability to capture distributed oscillatory patterns, with increased beta-band power during the abnormal event localized to sensorimotor regions showing concurrent increases in both beta and theta power (Fig. 11e). Analysis of the aperiodic exponent showed increased values during the abnormal discharge epoch (Fig. 11f), demonstrating the feasibility to observe changes in spectral characteristics across the cortical surface.

Together, these analyses demonstrate the array’s technical ability to capture transient abnormal discharges and resolve their spatial distribution across the cortical surface. The 32-channel surface array illustrates the scalability of this fabrication platform to high-density configurations enabling broad cortical coverage—an important translational consideration for applications such as clinical ECoG. While these recordings establish the array’s capability for spatial mapping of distributed cortical activity, comprehensive characterization of epileptic network dynamics would require larger cohorts and dedicated experimental protocols beyond the scope of the present study.

Overall, these recordings demonstrate that thin-film polyimide probes can provide stable, high-resolution monitoring of seizure activity across multiple days in awake, behaving animals. By using an epileptic model with spontaneous seizures, this study replicates the clinical context of sEEG, where depth electrodes are implanted for days to localize seizure onset zones. The findings highlight the translational relevance of this platform, showing that flexible thin-film depth probes can function effectively in a preclinical epilepsy setting and form the basis for future human-compatible sEEG devices.

While long-term chronic implantation studies are essential, the primary objective of this work is to establish a reproducible and tunable polyimide platform for neurosurgical interventions lasting less than 30 days. Many clinically relevant procedures such as sEEG, intraoperative MER, and functional cortical mapping require implantation for hours to weeks rather than permanent implantation. Accordingly, we validated the synthesized polyimide using depth, surface, and semi-chronic recordings in mice to mimic human neurosurgical workflows.

### E. Acute Systemic Toxicity Testing

ISO 10993-11 acute systemic toxicity testing was conducted on extracts prepared from the fabricated polyimide films, administered via intravenous and intraperitoneal routes in male Swiss Albino mice (N = 20). No mortality, abnormal clinical signs, or weight loss were observed over the 72 hr observation period; all animals showed physiological weight gain. Gross pathological examination revealed no abnormalities at injection sites or in major organs. These findings confirm that the synthesized BPDA–pPDA polyimide does not elicit acute systemic toxicity at the maximal extractable dose, supporting its safety for use in implantable neural devices and its potential for further preclinical and clinical translation.

ISO 10993-11 was the only biocompatibility assessment performed, as the scope of ISO 10993 includes multiple tests, such as cytotoxicity, sensitization, genotoxicity, implantation, and hemocompatibility, that are designed to evaluate the safety of a fully assembled medical device in its final clinical form. Polyimide films in isolation do not represent the complete device configuration, and testing them against the full suite of ISO 10993 standards would not yield meaningful or translationally relevant results. Factors such as the device’s geometry, encapsulation method, sterilization process, and intended anatomical contact duration all influence the overall biocompatibility profile and must be evaluated at the final product level. Therefore, the acute systemic toxicity study was chosen as an initial, material-level screen to establish baseline safety, with further device-specific testing planned once the polyimide is integrated into complete neural interface systems for preclinical and clinical translation.

Several studies have reported the successful use of polyimide-based neural probes, demonstrating favorable flexibility, and biocompatibility [18], [19], [20], [21], [22], [43], [44]. However, it is important to emphasize that these results were obtained using commercial polyimide formulations that, while effective for research use, are not classified under ISO 10993 or USP Class VI biocompatibility standards. Manufacturers of widely used polyimides such as PI-2611, PI-2555, and PI-2525 (HD Microsystems, DuPont) [45] and U-Varnish-S (UBE Industries) explicitly state that these materials are not designed, tested, or approved for medical or implantable use. Their proprietary compositions and lack of disclosure on residual solvents or additives make independent certification and regulatory clearance for human implantation infeasible. In contrast, the BPDA–pPDA polyimide synthesized in this study offers a transparent, controllable, and reproducible formulation with verified chemical safety (ISO 10993-11), establishing a credible foundation for translational development of clinically oriented thin-film neural interfaces.

## IV. Conclusion

Thin-film neural interfaces have historically faced translational barriers due to the lack of medical-grade, customizable polymer substrates. This work addresses that gap by introducing an open-source BPDA–pPDA polyimide platform that satisfies the thermal, chemical, and electrochemical requirements for flexible implantable electrodes, supported by ISO 10993-11 systemic toxicity validation and in vivo demonstrations in acute and semi-chronic epilepsy models. The platform enables high-quality neural recordings—including isolated single units and stable low-frequency LFPs—across clinically relevant implantation periods, replicating core features of sEEG monitoring. By overcoming supply-chain dependence and enabling tunability in film thickness, flexibility, and electrode geometry, the proposed material system establishes a practical route for advancing MEMS-based neural interfaces toward clinical adoption.

Despite these advances, long-term neural interfacing remains constrained by chronic foreign body response, marked by microglial activation, astrocytic encapsulation, increased impedance, and neuronal displacement that collectively degrade signal quality over weeks to months [40], [46], [47]. This response is driven largely by the mechanical mismatch between soft neural tissue (1–10 kPa) and conventional rigid substrates (50–200 GPa), compounded by micromotion-induced strain at the tissue–device interface [48]. Emerging strategies—including flexible polymers, bioresorbable shuttles, anti-inflammatory coatings, and optimized geometries—highlight the importance of mechanical compliance alongside electrochemical performance [49], [50]. As chronic performance metrics evolve through standardized impedance, histological, and functional benchmarks [51], [52], [53], [54], the flexible and tunable BPDA–pPDA substrate developed here offers a foundation for further development toward chronically stable neural probes. By enabling precise mechanical tailoring and reliable recording performance, this platform advances the development of chronically stable neural interfaces for long-term neuroscience research and clinical neuroprosthetic applications.
